# Supplementary material for: Characterization of SSR genomic abundance and identification of SSR markers for population genetics in Chinese jujube (Ziziphus jujuba Mill.)
Source: PeerJ. 2016 Feb 22;4:e1735. doi: 10.7717/peerj.1735 (PMC4768703; doi:10.7717/peerj.1735)
Supplement: Table S1 [file peerj-04-1735-s001.docx]

| Species | Cultivar name | Sample No. | Orgin |
| --- | --- | --- | --- |
| *Z. acidojujuba* |  | Fu2015017 | Luoyang, Henan |
| *Z. jujuba* | Bianhesuan | Fu2015019 | Luoyang, Henan |
|  | Huizao | Fu2015020 | Luoyang, Henan |
|  | Xiaobaizao | Fu2015024 | Xinzheng, Henan |
|  | Hupingzao | Fu2015025 | Xinzheng, Henan |
|  | Damaya | Fu2015026 | Xinzheng, Henan |
|  | Dongzao | Fu2015027 | Xinzheng, Henan |
|  | Jixinzao | Fu2015028 | Xinzheng, Henan |
|  | Yuanling | Fu2015029 | Xinzheng, Henan |
|  | Longzao | Fu2015031 | Xinzheng, Henan |
|  | Tailihong | Fu2015033 | Xinzheng, Henan |
|  | Zanhuangdazao | Fu2015035 | Xinzheng, Henan |
|  | Chahuzao | Fu2015036 | Xinzheng, Henan |
